# Smart furniture using radar technology for cardiac health monitoring

**DOI:** 10.1038/s41598-024-80062-5

**Published:** 2025-01-09

**Authors:** Ali Gharamohammadi, Mohammad Omid Bagheri, Serene Abu-Sardanah, Michael M. Y. R. Riad, Hajar Abedi, Ahmad Ansariyan, Kang Wang, Ashish Saragadam, Dmytro Chumachenko, Shahabeddin Abhari, Plinio Pelegrini Morita, Amir Khajepour, George Shaker

**Affiliations:** 1 Department of Mechanical and Mechatronics Engineering, University of Waterloo, Waterloo, ON Canada; 2 Department of Electrical and Computer Engineering, University of Waterloo, Waterloo, ON Canada; 3 School of Public Health Sciences, University of Waterloo, Waterloo, ON Canada; 4 Department of Mathematical Modelling and Artificial Intelligence, National Aerospace University, Kharkiv Aviation Institute, Kharkiv, Ukraine

**Keywords:** Biomedical engineering, Electrical and electronic engineering

## Abstract

The integration of radar technology into smart furniture represents a practical approach to health monitoring, circumventing the concerns regarding user convenience and privacy often encountered by conventional smart home systems. Radar technology’s inherent non-contact methodology, privacy-preserving features, adaptability to diverse environmental conditions, and high precision characteristics collectively establish it a compelling alternative for comprehensive health monitoring within domestic environments. In this paper, we introduce a millimeter (mm)-wave radar system positioned strategically behind a seat, featuring an algorithm capable of identifying unique cardiac waveform patterns for healthy subjects. These patterns are characterized by two peaks followed by a valley in each cycle, which can be correlated to Electrocardiogram (ECG), enabling effective cardiac waveform monitoring. The provided algorithm excels in discerning variations in heart patterns, particularly in individuals with prolonged corrected QT intervals, by minimizing high frequency breathing interference and ensuring accurate pattern recognition. Additionally, this paper addresses the influence of body movements in seated individuals, conducting a comprehensive study on heart rate variability and estimation. Experiment results demonstrate a maximum interbeat intervals (IBI) error of 30 milliseconds and an average relative error of 4.8% in heart rate estimation, showcasing the efficacy of the proposed method utilizing variational mode decomposition and a multi-bin approach.

## Introduction

Smart home monitoring is recognized as a key application of health sensors and internet of things, offering advantages in diagnostics, long-term monitoring, and emergency event detection. Among various applications, vital sign monitoring in smart furniture stands out as a critical means of assessing residents’ health status, providing essential information such as breathing rate (BR), heart rate (HR), and heart rate variability (HRV)^1–11^.

The global population has witnessed a growing incidence of fatalities attributed to chronic and cardiovascular diseases^12,13^. Electrocardiography and echocardiography are established standards for monitoring heart activity. ECGs track the heart’s electrical activity, while echocardiograms provide insights into the heart’s structure and blood vessels^14,15^. These diagnostic tools are effective for detecting cardiovascular diseases but come at a significant cost and require specialized training to operate. Frequency modulated continuous wave (FMCW) radar, a highly motion-sensitive sensor, offers a cost-effective and user-friendly alternative for certain conditions. Despite operating at millimeter wavelengths with limited skin penetration^16^, FMCW radar can still detect skin displacements caused by heartbeats. By correlating these displacements with the ECG signal, which associates heart motions with P, Q, R, S, and T-waves, employing electrical cardiac insights in conjunction with radar sensors is possible. However, recent research has predominantly concentrated on HR estimation^17–21^ rather than cardiac waveform monitoring, which can be used to extract HRV. HRV metrics, such as the root mean square of successive differences (RMSSD), the standard deviation of the RR interval (SDRR), and the percentage of successive IBIs that differ by more than 50 ms (pNN50) can be derived to assess the accuracy of HRV estimation.

One of the most recent insights is to estimate HRV which refers to the fluctuations in the time intervals between successive heartbeats, also known as IBIs. Accurate HRV monitoring plays a crucial role in various applications, including the early diagnosis of cardiovascular disorders, stress assessment, recognition of emotions, and anxiety treatment^22–24^. Estimating HRV from radar signals provides a valuable opportunity for deeper insights.

More recently, some studies have investigated the estimation of cardiac waveform using radar signals which can be used for HR and HRV estimation. Some of these studies have utilized neural networks and machine learning techniques to derive ECG patterns from radar data^14,25–31^. However, these methodologies are predominantly trained on data from individuals without cardiovascular conditions. Consequently, their applicability to individuals with heart diseases may be uncertain. The correlation between cardiac waveform from radar and ECG signal reveals that the T-peak in the ECG signal corresponds to a downward peak in the estimated displacement by radar; however, these findings were observed during a breath-hold period without body movements^15^.

Other studies employed wearable radars to reconstruct cardiac waveform^32–35^. The literature presents a quadruple radar system designed to monitor each main chamber of the heart using a node^32,33^. The results indicate that the waveforms of those nodes are highly correlated when their signals are not blocked. Consequently, the study focusing on estimating the cardiac waveform using a single radar is presented^34^. Although wearable radar systems have an accurate HRV estimation, the proposed setup is unfeasible for real-world scenarios since they must be worn all the time. Therefore, it is crucial to use these systems for real-world scenarios.

Prolonged corrected QT intervals (QTc) have been identified as a risk factor contributing to sudden cardiac deaths in the general population^36^. Therefore, monitoring individuals with this heart condition is extremely valuable. Recent studies mostly investigated their algorithms in healthy subjects for HR and HRV estimation^22,27,34,37–42^. However, individuals with prolonged QTc may have normal HR and HRV^36^.

Three major challenges in real-world smart furniture scenarios need to be addressed: high frequency harmonics of breathing, body movements, and range bin selection. In many studies reported in the literature, participants were asked to hold their breath or breathe shallowly while seated^15,32,34,38,40,43^. Some signal processing approaches are also employed to remove high frequency harmonics of breathing and compensate body movements^37,41,44^. However, range bin selection is still a challenge in these investigations.

This paper presents a solution for addressing challenges associated with smart furniture by leveraging mm-wave radar technology, with the following contributions: I) Investigations on the cardiac waveform of healthy subjects by radar signals reveal that heart displacements on the skin have two peaks sequenced with one valley in each cycle. This pattern is verified in different breathing situations, including shallow and deep breathing patterns. Additionally, the investigation of this pattern extends to different subjects while HR was high or low. Based on the reconstructed cardiac waveform, HR and HRV are estimated; II) In individuals with the prolonged QTc condition, the previously mentioned typical cardiac waveform pattern is no longer applicable. Consequently, this distinctive cardiac waveform can serve as an effective indicator for detecting heart-related issues. We have confirmed this distinction through validation with multiple subjects exhibiting the prolonged QTc condition; III) Investigation on the deep and shallow breathing removal from heart displacements using a heuristic approach that utilizes the VMD algorithm for harmonic analysis. Our findings indicate that, even in the presence of deep breathing, it is feasible to reconstruct the cardiac waveform of healthy individuals which has two peaks sequenced with one valley in each cycle using a minimum of two harmonics. It is essential to note that if the identified harmonics for cardiac waveform reconstruction correspond for the second, third, or fourth harmonics of breathing, they should be excluded from the cardiac waveform reconstruction process; IV) Investigations on different body movements of seated people. The results indicated that body movements usually cause spikes in estimated movements by radar signal. These movements can introduce interference across a wide range of frequency bands, covering both breathing and heartbeat frequencies. Therefore, it is concluded that these movements cannot be compensated by frequency domain approaches like filtering. Consequently, it is imperative to employ time-domain approaches to effectively address and mitigate the interference caused by these movements. In addition, if body movement compensation cannot recover a range bin, the corrupted range bin is removed by comparing with other range bins. V) Positioning the proposed radar system behind the seat. This sensor placement eliminates the need for any range bin selection in the signal processing chain. Additionally, noise effects on the phase become more pronounced at longer distances compared to closer ranges.

## Methods

### Cardiac anatomy and electrical activity in healthy individuals

**Fig. 1:**
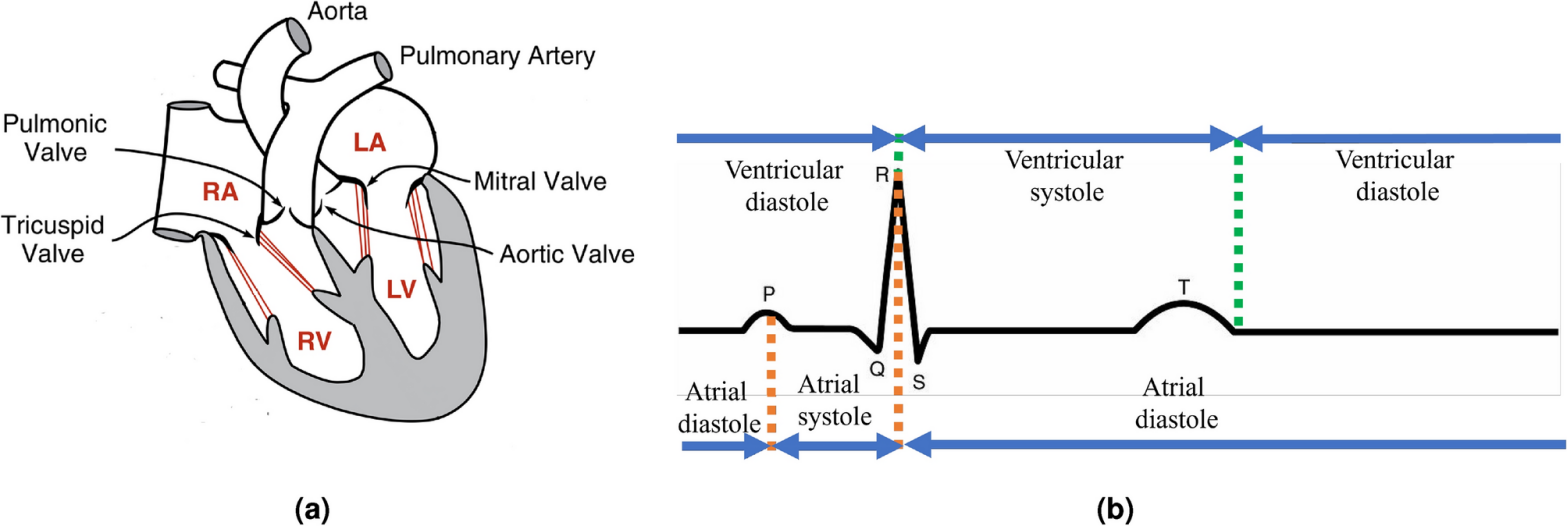
Cardiac physiology and function: (**a**) Anatomy of the heart^44^ and (**b**) ECG waves and their relation to the cardiac cycle.

The heart is typically divided into four main chambers, the right atrium (RA), right ventricle (RV), left atrium (LA), and left ventricle (LV) as shown in Fig. 1a. The atria and ventricles are divided by atrioventricular valves, while the left and right ventricles are separated by the ventricular septum. The left ventricle is linked to the aorta through the aortic valve, and the right ventricle is connected to the pulmonary artery through the pulmonary valve^45^.

In a healthy individual, the heart’s electrical and mechanical activities follow a specific pattern. It starts with an electrical signal, the depolarization wave, originating in the right atrium, leading to atrial contraction (P wave on the ECG). This atrial contraction is known as atrial systole. The depolarization of the right and left ventricles (marked by the QRS complex on the ECG) triggers their contraction, initiating ventricular systole. Repolarization of the ventricular muscle cells occurs in two phases, spanning from the end of the QRS complex to the end of the T wave. The cardiac cycle is completed with the T wave. Because the ventricles are larger and depolarize more quickly than the atria, their contraction during ventricular systole is more forceful and rapid^45^. The key stages of the cardiac cycle and their alignment with ECG waves are illustrated in Fig. 1b.

### Fundamental of FMCW radar for vital sign monitoring

**Fig. 2:**
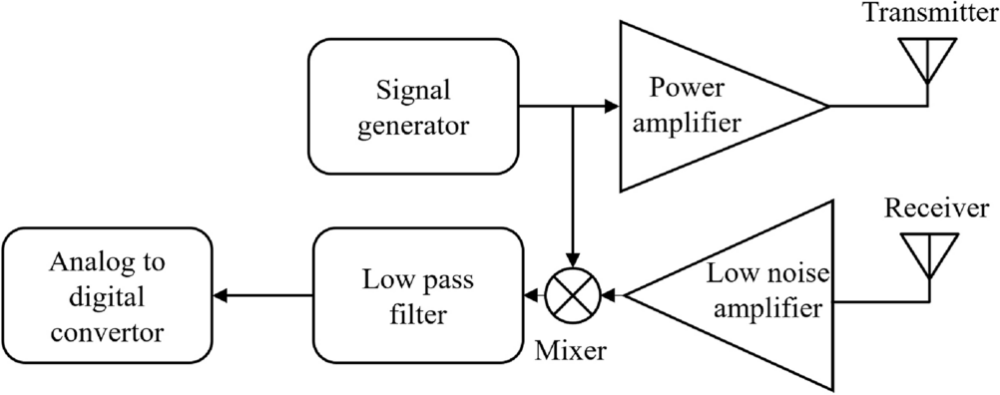
The block diagram of radar system before digital signal processing.

The fundamental principle underlying the FMCW Radar system is founded upon the transmission of an amplified electromagnetic signal, originating from a signal generator, into the surrounding environment. Subsequently, the radar system captures reflected signals back from different objects, conveying object properties such as distance and velocity. Figure 2 represents the block diagram of a radar module, wherein both the transmitter and receiver coexist within the same physical location. Following the amplification of the received signal through the utilization of a low-noise amplifier, a mixer correlates the transmitted and received signals, generating both low-frequency and high-frequency signals. To effectively eliminate the high-frequency components, the system incorporates a low-pass filter. Subsequently, an analog-to-digital converter is employed to convert the analog signal into a digital format^46^.

In FMCW radar, the signal generator performs a sweep from the starting frequency, denoted as $f_{min}$, to the final frequency, denoted as $f_{max}$, with a positive slope represented as *K*. The generated signal from this sweep is called chirp. FMCW radars can accommodate a specific range of chirp slopes. Therefore, it is important to design the chirp slope within a range that is achievable and practical. This frequency sweep takes place over a specified time duration, denoted as *T*. The resulting signal generated by the signal generator is commonly referred to as a chirp. The resultant frequency bandwidth (BW) for a chirp is determined as (1),

1 $$\begin{aligned} BW = f_{\max } - f_{\min } = KT \end{aligned}$$

The frequency bandwidth also plays a crucial role in determining the range resolution of the radar. The relationship between range resolution and frequency bandwidth is expressed as (2),

2 $$\begin{aligned} \Delta R = \frac{c}{2 \cdot BW} \end{aligned}$$

where $\Delta$*R* and *c* are range resolution and light velocity in free space respectively. Range resolution discretizes the range in FMCW radar^47^. Improved range resolution results in more precise range estimation and enhanced differentiation between closely spaced reflections. In FMCW radar, the range can be estimated by identifying the peak frequency of the reflected chirp signal in the frequency domain. The transmitted chirp signal (*x*(*t*)) in the time domain can be expressed in (3). Suppose that there is a single reflection with a delay of $t_{d}$ from the environment. Then, the resulting output of the low pass filter (*y*(*t*)) is calculated as (4). where $\alpha$ represents the influence of both the environment and the target on the amplitude of the transmitted signal and $t_{d}$ is calculated as $t_{d}$=(2*R*)/*c*. This low pass signal is called beat signal. Given the low range of the target, $t_{d}$ is significantly less than t. Consequently, the beat signal can be simplified as (5). As the last step, the range of the target can be determined by applying a fast Fourier transform (FFT) to the low pass signal.

3 $$\begin{aligned} x(t) = A \cos (2\pi . f_{\text {min}}. t + \pi . K. t^2), \quad 0< t < T \end{aligned}$$

4 $$\begin{aligned} y(t) = A^2. \alpha . \cos \left( 2\pi . f_{\text {min}}. t_d + 2\pi . k \left( t.t_d - t_d^2\right) \right) \end{aligned}$$

5 $$\begin{aligned} y(t) \approx A^2. \alpha . \cos (2\pi . f_{\text {min}}. t_d + 2\pi . k.t. t_d) \end{aligned}$$

### Signal and system design for vital sign monitoring

There are several factors that can be considered for sensor selection. Firstly, radar with higher carrier frequencies has low levels of noise. Secondly, higher carrier frequency has higher sensitivity to small movements. Therefore, radar with higher carrier frequencies can monitor small movements more accurately^48^. There is an extensive study on different radars with different center frequencies, including 7.7, 24, and 77 GHz^41^. The results indicate that radar with higher frequency has less estimation error than other radars. However, the federal communications commission (FCC) is currently prioritizing the use of the 60 GHz band for health applications and indoor use cases^49^. As a result, an off-the-shelf 60 GHz radar is used in this study.

In signal design, several parameters directly influence the system’s functionality like pulse repetition frequency, range resolution, number of samples per chirp, number of chirps in a frame, sampling rate, chirp slope, and idle time. Table 1 summarizes the designed signal’s parameters. The radar can produce a couple of frames per second which is known as the pulse repetition frequency. The pulse repetition frequency can be designed based on the maximum frequency of vibrations. 6 Hz is an acceptable boundary for cardiac waveform monitoring^34^. However, in this paper, 10 Hz is considered the maximum frequency of vibration. Hence, the pulse repetition frequency is 20 based on Nyquist theorem.

**Table 1:** Designed signal parameters.

Parameter	Value
Pulse repetition frequency	20 Hz
Bandwidth	5 GHz
Range resolution	3 cm
Maximum range	96 cm
Number of samples	64
Sampling rate	1 MHz
Chirp slope	78.128 MHz/$\mu$s
Idle time	5 $\mu$s
Number of chirps	32

Another effective parameter which can improve results is range resolution^48^. In this paper, the BGT60TR13C FMCW radar from Infineon Technologies is used. This sensor can produce an FMCW signal from 58 GHz to 63.5 GHz modulated by the sawtooth wave. The achievable range resolution based on the provided documents from Infineon is almost 3 cm^50^. As a result, the effective bandwidth is 5 GHz. The number of samples can determine the maximum achievable range by considering the range resolution of the system.

The number of chirps is another factor that has a direct effect on signal quality. Since breathing and heart displacements are slow activities, the reflected chirps in a pulse are very similar to each other. However, by taking an average from all the chirps in a pulse, the phase noise of the signal can be improved. In this paper, we have considered 32 chirps per pulse. Other parameters are chosen accordingly^51^.

**Fig. 3:**
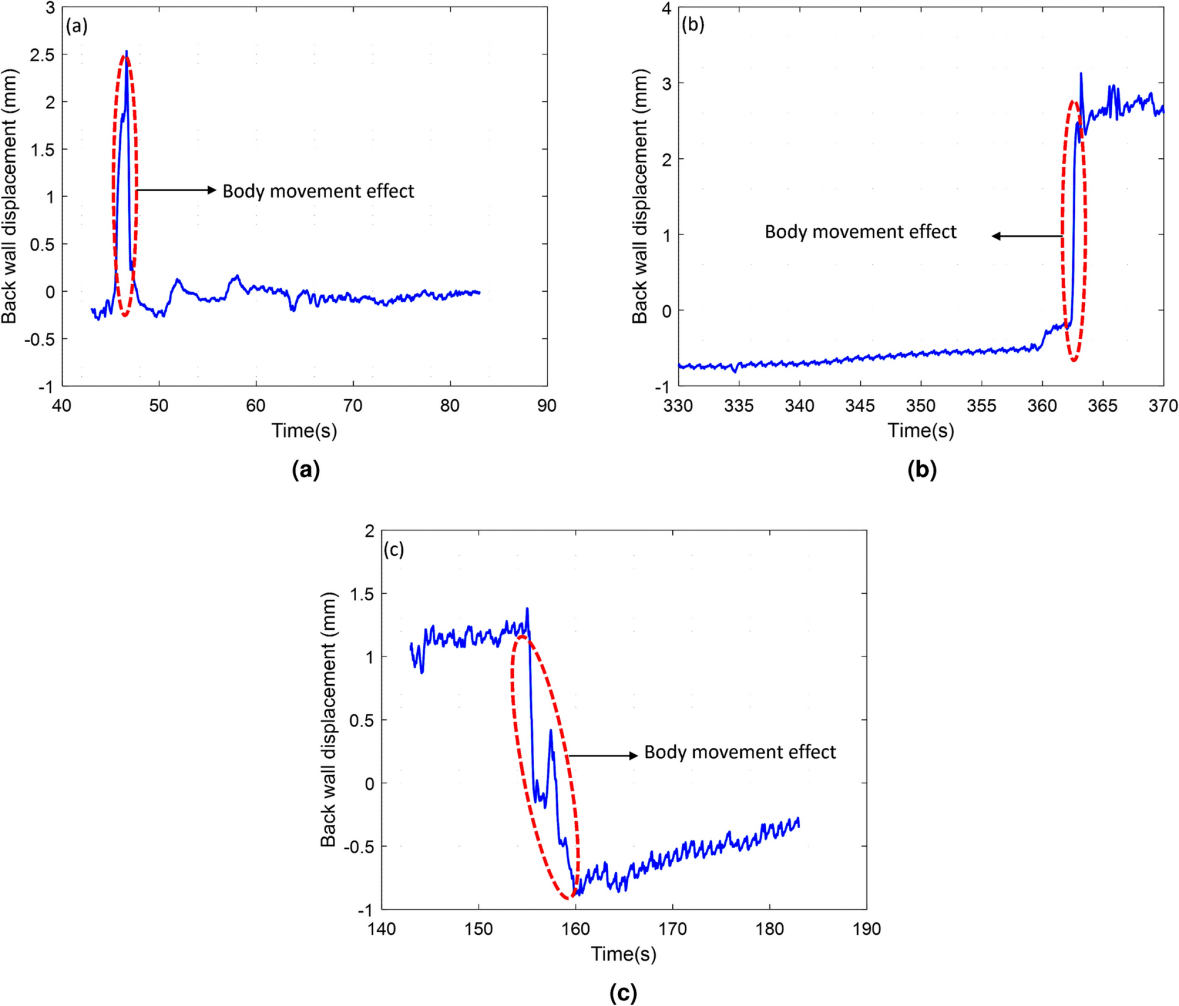
Torso movements’ impact on the unfiltered vibration signal: (**a**) narrow strong pulse (**b**) positive DC offset (**c**) negative DC offset.

### Undesired harmonics and body movement effects

The first step to accurately monitor cardiac waveform is to understand other movements, including breathing and body movements. Firstly, it is necessary to estimate BR since breathing harmonics have a comparable amplitude to the cardiac waveform. If the cardiac HR peak, typically in the range of 0.8-6 Hz, is determined to be an integer multiple of the BR frequency, the HR value and resulting waveform, cannot be trusted. In addition, the intermodulation harmonics can be generated because of the non-linear relation between breathing and heart displacements. These harmonics are not stronger than the cardiac waveform since the effect of each displacement is based on the Bessel function of the first kind which is less than one^52^.

Secondly, body movements have a direct effect on the measurement of breathing and cardiac waveform. Since these movements usually have greater displacements than breathing and heart displacements, they can corrupt human vibration signals in the frequency domain. Figure 3 shows the torso movements’ effect on the unfiltered vibration signal when the radar is behind a seat. As can be seen in Fig. 3a, the body movement effect is similar to a narrow strong pulse. Therefore, its frequency response is similar to a sinc function with strong low frequency components, which can make BR and HR estimations difficult. In addition, since it has a strong amplitude, it can generate strong high frequency components that might affect estimated cardiac waveform as well. Figure 3b,c show positive and negative DC offsets respectively. These DC offsets can affect all the frequencies and can make BR, HR, breathing waveform, and cardiac waveform.

**Fig. 4:**
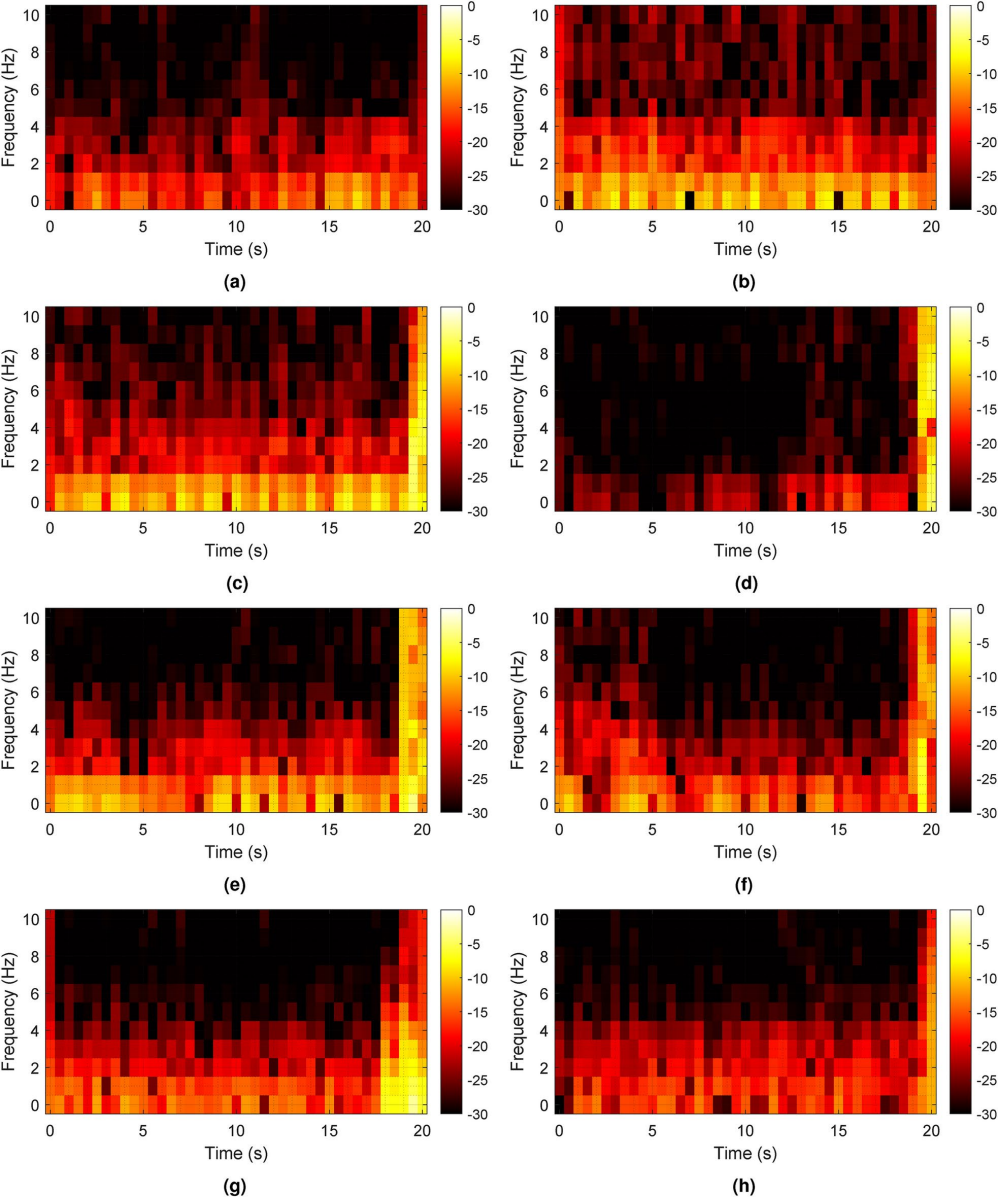
The STFT of different activities in which the vibration signal is filtered by a high pass filter with a cut-off frequency of 0.1 Hz: (**a**) normal breathing (**b**) deep breathing (**c**) standing (**d**) sitting (**e**) torso movements (**f**) random body movements (**g**) hitting the chair (**h**) swinging the chair.

Figure 4 depicts the STFT of different activities in which the signal is filtered by a high pass filter with a cutoff frequency of 0.1 Hz. In all the measurements except sitting, the participant was breathing. Normal and deep breathing do not exhibit significant high-amplitude components, as demonstrated in Fig. 4a,b, respectively. However, other activities in which torso moves forward or backward, as shown in Fig. 4c–h, have high-amplitude displacement and can make strong frequency components, especially in lower frequencies. Therefore, body movements can distort breathing and heart displacements. As a result, since these body movements cannot be mitigated by filtering and can affect BR and HR estimations, they should be compensated in the time domain. By taking a derivative from the unfiltered signal and setting a threshold for the spikes, those spikes can be detected and compensated. The threshold can be determined by studying different participants with different breathing rates and depths.

### Signal processing chain to reconstruct cardiac waveform

**Fig. 5:**
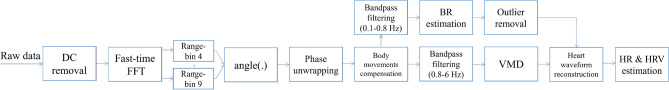
The signal processing chain to reconstruct cardiac waveform from human body vibrations.

Figure 5 depicts the signal processing chain to reconstruct cardiac waveform from human body vibrations. In this paper, after DC removal and fast-time FFT, multiple range bins are employed to reconstruct the cardiac waveform. Since the torso width is more than 40 cm for adults^53^, and radar is placed in the middle of the back, 6 range bins (from range bin number 4 to 9) are chosen, considering a 3 cm range resolution^51^. Then, phase of each bin is extracted to reconstruct body vibrations. Due to the high amplitude of human body vibrations in comparison to wavelength, which is almost 5 mm, it is necessary to unwrap the phase.

Multiple steps were employed to mitigate the high amplitude body movements in the time domain as provided in Algorithm 1. Firstly, the derivative of the signal is taken to find spikes in the vibration signal. In the next step, a threshold is used to determine the spikes in the signal. The threshold level determination needs to be done statistically by collecting data from different participants. These participants were asked to breathe shallowly and deeply while seated. Approximately 300 measurements were collected from a group of 4 participants to estimate statistical parameters. The threshold level based on the empirical rule^54^, is determined as 0.48 mm/sample. If the amplitude of the differentiated movement is more than this threshold, the derivative of the signal is set to zero. Finally, the cumulative sum in Algorithm 1 reconstructs the body vibration signal with compensated body movements.

**Algorithm 1:**
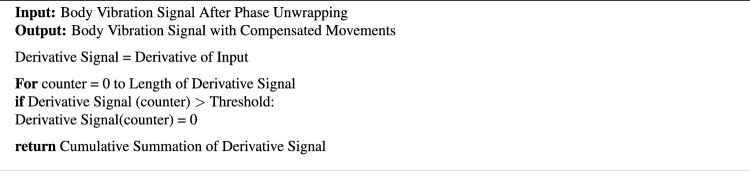
Body movement compensation

**Algorithm 2:**
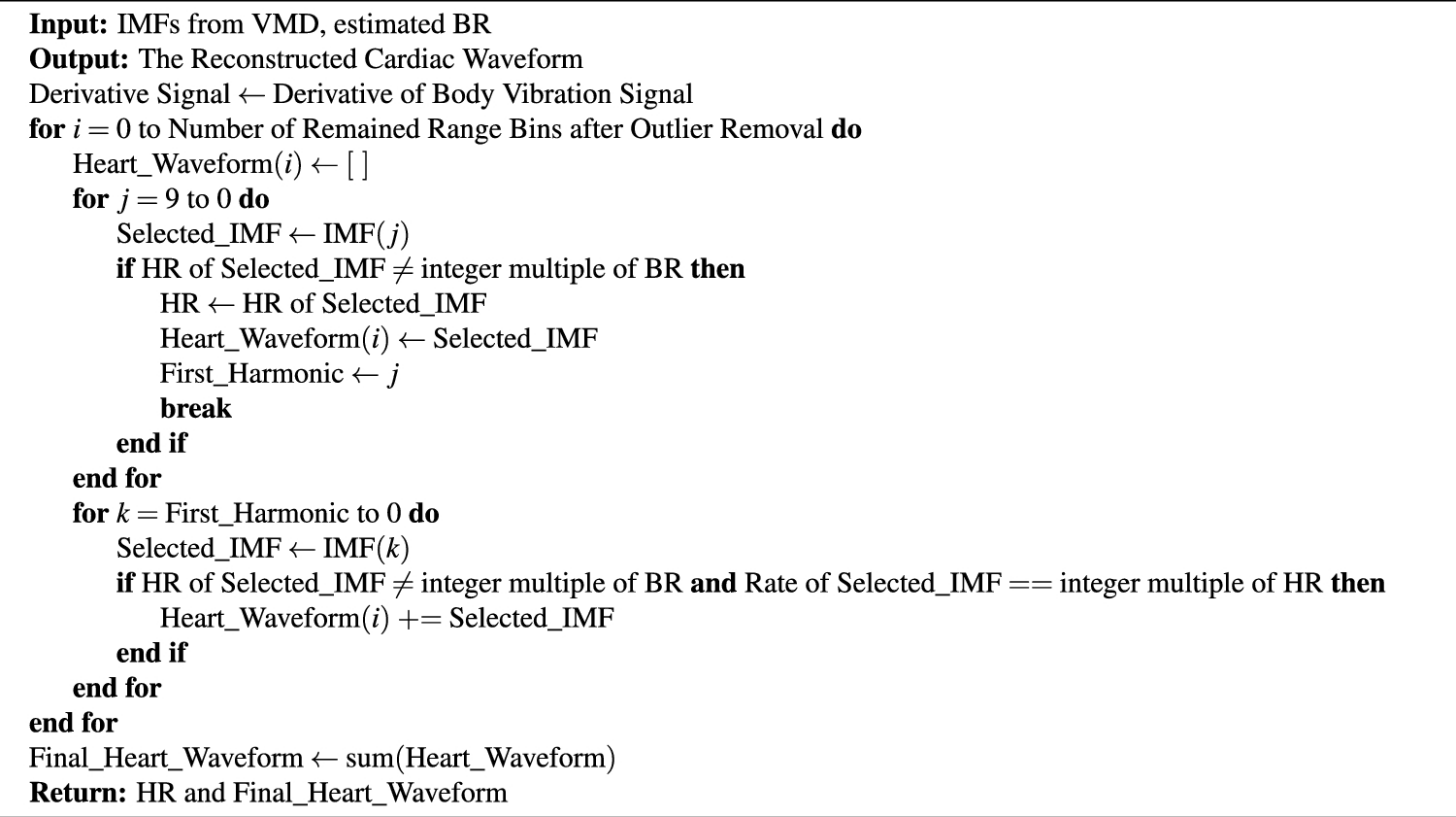
Reconstruction of cardiac waveform from VMD IMFs and estimated BR

After body movement compensation, the body vibration signal can be used for BR, HR, and HRV estimations. Since the breathing waveform is the second interfere after body movement compensation for cardiac waveform reconstruction, it should be excluded from cardiac waveform reconstruction. Due to the periodic nature of breathing, BR estimation can determine breathing harmonics. Therefore, the body vibration signal without body movements is filtered by a band pass filter from 0.1 Hz to 0.8 Hz. In addition to this human body movement compensation, an outlier removal is applied to remove distorted range bins. In this paper, the one-sigma approach in outlier removal considers data points within one standard deviation from the mean as normal and excludes those outside this range as outliers.

To estimate HR and HRV, the main harmonic of the breathing waveform can be filtered by a band pass filter from 0.8 Hz to 6 Hz. A narrower bandpass filter removes cardiac waveform details. However, higher frequency harmonics of breathing waveform still can interfere with cardiac waveform, especially during deep breathing. VMD approach can be employed to decompose different harmonics after band pass filtering. In this paper, the filtered signal is decomposed into 10 intrinsic mode functions (IMF). The strongest IMF, which is not an integer multiple of BR, is the main harmonic of the cardiac waveform. As we discussed earlier, nonlinear harmonics generated from breathing and heart displacements cannot be stronger than the main harmonic of heart. If the higher harmonics of the cardiac waveform are not an integer multiple of BR, they can be used for cardiac waveform reconstruction. The cardiac waveform should be reconstructed by at least two harmonics. Otherwise, the cardiac waveform cannot be reconstructed. Finally, HRV can be estimated based on the reconstructed waveform. Algorithm 2 provides the cardiac waveform reconstruction.

### Experimental setup and protocol for radar-based health monitoring

In this study, we employed the BGT60TR13C radar module developed by Infineon. This radar module operates at 60 GHz and can provide an almost 5 GHz bandwidth. The overall dimensions of this radar package measure 64 mm $\times$ 25.4 mm^55^. Due to its compact size, this radar module can be integrated into smart furniture without causing any inconvenience to occupants.

The placement of the sensor significantly affects the radar’s ability to measure displacement accurately. There are two key factors to consider in sensor placement. First, the angle and distance between the radar and the human body play a crucial role. Placing the radar in front of the displacement enables precise estimation because the radar measures radial displacements, and high-amplitude displacements are less susceptible to noise interference. Moreover, the radar equation shows that reflected power increases significantly with shorter distances to the fourth power, making radar measurements less susceptible to noise at close ranges. Therefore, for the most precise displacement estimates, it is advisable to position the radar in front of the displacement at the closest possible range.

**Fig. 6:**
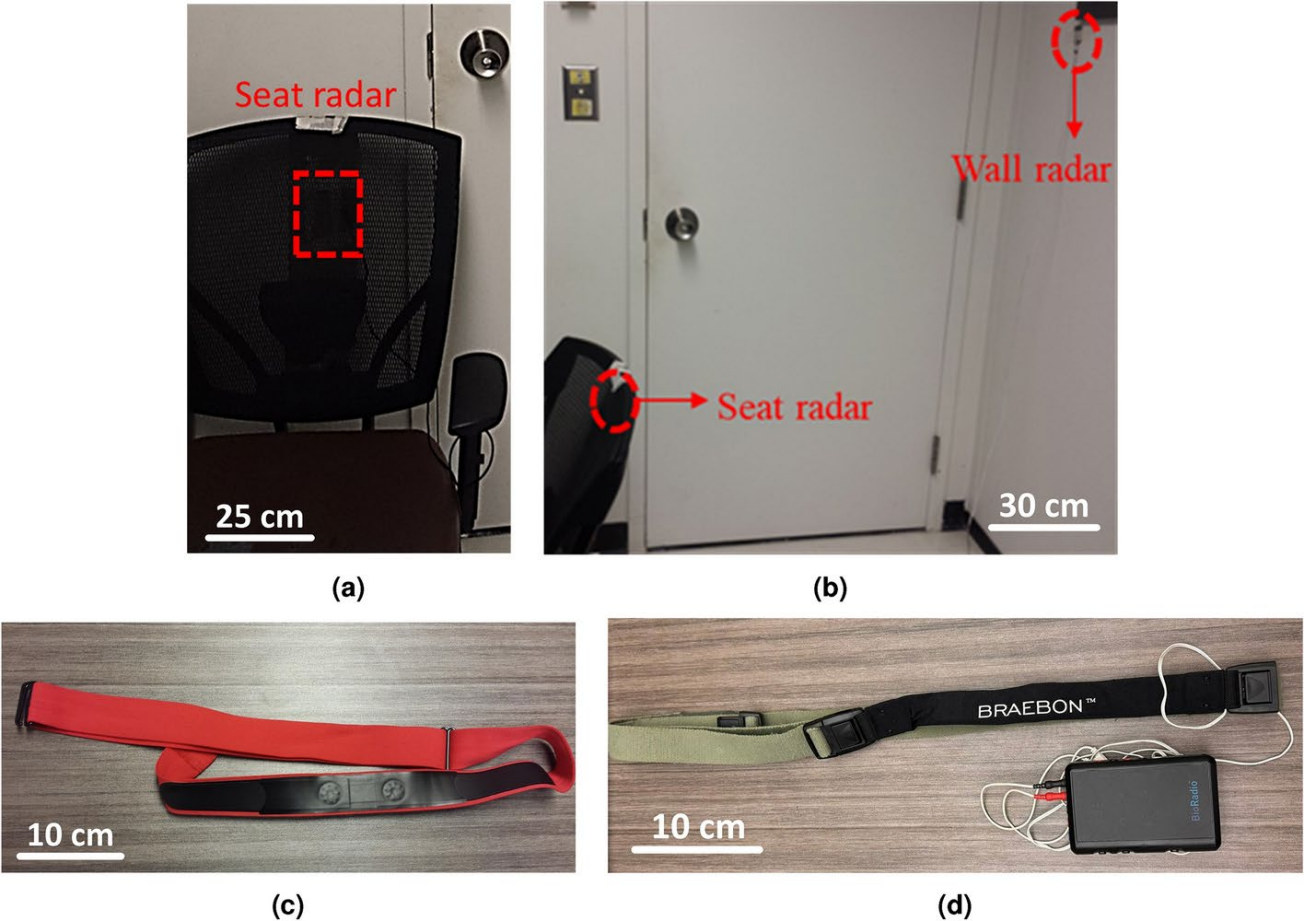
The experimental setup: (**a**) the experimental setup with the seat radar installed behind the seat. (**b**) the interference test setup in the presence of wall radar. (**c**) Frontier X for ECG data collection. (**d**) BioRadio piezo electric respiratory effort belt for respiratory data collection.

Secondly, for individuals in a seated position, the radar can be placed either on the desk in front of the chest or behind the seat. The desk-mounted radar can effectively measure chest and abdomen wall displacements, which are more pronounced than back wall displacement. However, it may be more susceptible to interference from other human body movements, such as hand and head movements. In contrast, the seat-mounted radar operates at a closer range, resulting in a stronger signal-to-noise ratio. Furthermore, it offers a more stable setup than the desk-mounted radar, which can be easily altered due to its position on the desk. In addition, the results obtained from the seat radar can be compared with those from a setup where the radar is attached to the shirt using adhesive tape. Finally, the effect of different radars in a smart home is also explored in terms of interference while those radars are synchronized or non-synchronized. Finally, the impact of different radars in a smart home is also examined concerning interference, considering whether those radars are synchronized or non-synchronized. Figure 6 illustrates the experimental setup for data collection. Participants were asked to sit on a chair and were given the option to either work with their laptops or take a rest. Figure 6a depicts the seat radar for data collection, installed behind the seat. Figure 6b depicts the experimental setup for interference effect from wall radar with and without synchronization. To evaluate seat radar results, two different belts, including Frontier X for ECG data collection^56^, as can be seen in Fig. 6c and BioRadio piezo electric respiratory effort belt for respiratory data collection^57^, as can be seen in Fig. 6d, are employed.

In the first data campaign, the accuracy of HRV and HR estimation are evaluated. Eight healthy subjects (four females and four males; age: 30 ± 11 years; weight: 70 ± 20 kg; height: 164 ± 14 cm) participated in these experiments. The duration of the main measurement was around 20 mins. Additionally, we also investigated body movements detection in human body vibrations in a separate measurement.

In the second data campaign, the evaluation focused on studying the cardiac waveform in older adult subjects with and without cardiac conditions. The subject group consisted of 15 subjects with an average age of 75 years (±5 years), average weight of 65 kg (±10 kg), and average height of 167 cm (±9 cm). These individuals actively engaged in the experiments while in a seated position, and the measurement duration was approximately 12 minutes. Notably, among these participants, five were identified with heart issues based on ECG data. Specifically, one participant exhibited Tachycardia, while the remaining four had a prolonged QTc. The University of Waterloo’s Clinical Research Ethics Committee approved the study for data collection (ORE #: 43843). All participants provided informed consent for their data to be collected, analyzed, and for the results of the study to be published. All methods were carried out in accordance with relevant guidelines and regulations. The raw individual data files during the current study are not publicly available because explicit consent for sharing individual data was not obtained from the participants. However, while we cannot share specific data from individual participants, we can provide detailed descriptions of the data collected and summarize the findings and insights gained from each participant’s data.

## Results

### Cardiac waveform verification

**Fig. 7:**
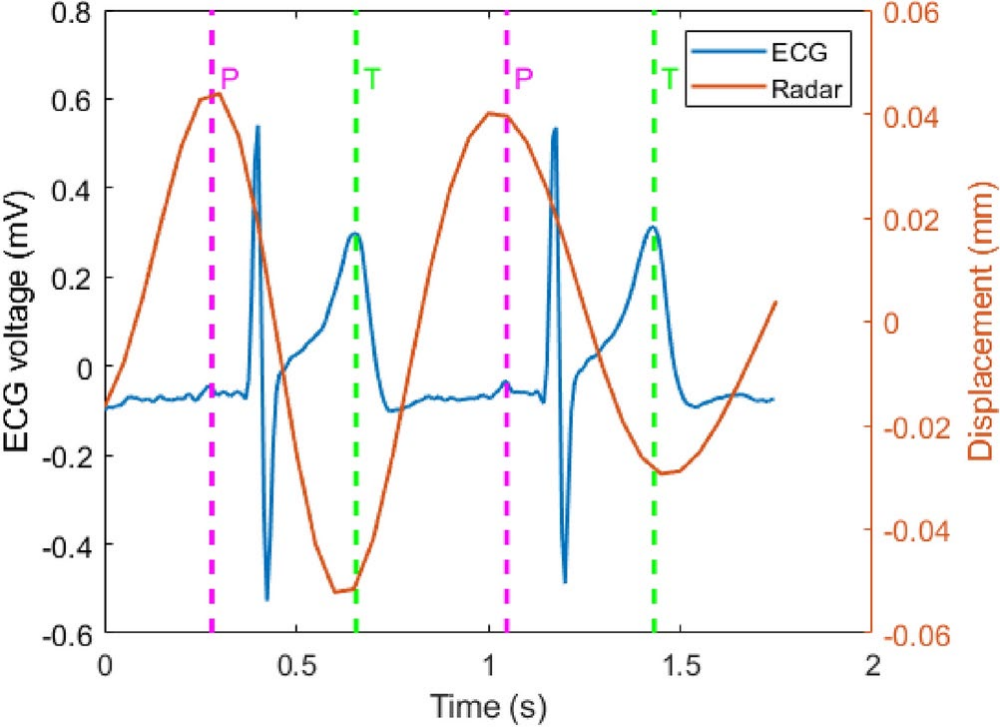
Cardiac waveform filtered from 0.8 Hz to 3 Hz in a breath-hold period compared with ECG data. The green and pink dashed lines correspond for T and P-waves, respectively.

In this paper, our investigations of radar signals measuring cardiac waveform indicate a periodic pattern on the skin. Figure 7 represents cardiac waveform filtered from 0.8 Hz to 3 Hz in a breath-hold period, synchronized with ECG data. In this measurement, the radar is placed in the middle of the back of the seat and its z-height is equal to heart height. It is concluded that T-waves correspond for valleys in radar displacements after synchronization^15^, which is compatible with our findings in this paper. Figure 7 also shows that P-waves can correspond for the peak value of displacements. However, wider bandwidth might be required for more details^34^.

**Fig. 8:**
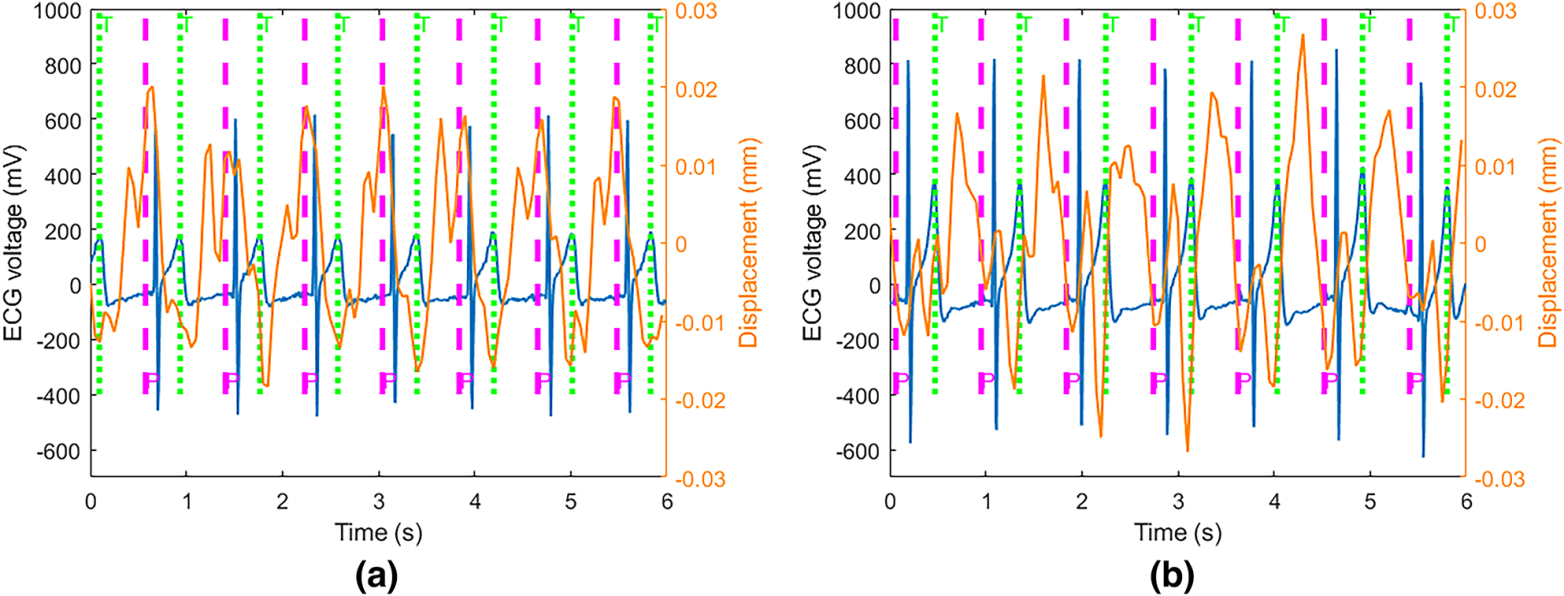
The cardiac waveform filtered from 0.8 to 6 Hz while radar has the same height as heart during a breath-hold in two distinct setups: (**a**) radar is located behind the seat and (**b**) radar is located on the chest and attached to shirt by an adhesive tape. The green and pink dashed lines correspond for T and P-waves, respectively.

Figure 8a represents the cardiac waveform filtered from 0.8 Hz to 6 Hz while the radar is located behind the seat and has the same height as heart during a breath-hold. Each heart cycle by radar signals has two sequential peaks and one valley. If valleys correspond for T-waves, the second peaks correspond for P-waves. Furthermore, when the participant is in a seated, calm state, the second peak is predominantly stronger than the first peak. However, when the radar is located on the chest and attached to the shirt by an adhesive tape^34^, the second peaks do not correspond for P-waves in cardiac waveform with the same filtering as can be seen in Fig. 8b. Additionally, our investigation into the influence of radar height revealed that similar cardiac waveforms are obtained when the radar height is within the range of HH +5 to HH -10, where HH represents heart height.

**Fig. 9:**
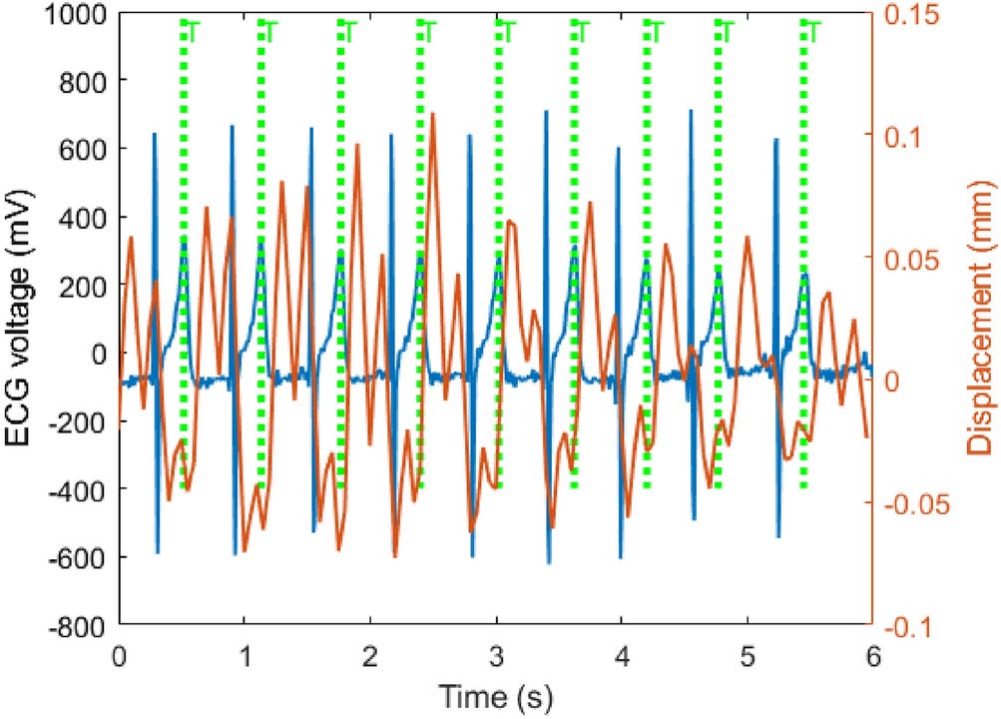
The cardiac waveform of a participant during a breath-hold followed an exercise, with the radar positioned behind the seat at chest height. The green dashed lines correspond for T-waves.

Figure 9 represents the cardiac waveform of a participant during a breath-hold after a jugging while the radar is located behind the seat at almost the same height as the heart. The cardiac waveform pattern is similar to a cardiac waveform during a calm state although HR is 98 beats per second. However, the first peak is stronger than the second peak in the heart displacements. In addition, the amplitude of cardiac waveform is almost 7 dB more than during a calm state which is a a physiological response to physical activities. Furthermore, P-waves in this measurement do not correspond to the second peak when T-waves are aligned with valleys. As a result, although cardiac waveform is a quasi-periodic signal, its valley can be reliably used for HRV estimation in different radar locations.

### Validation of the proposed radar setup

**Fig. 10:**
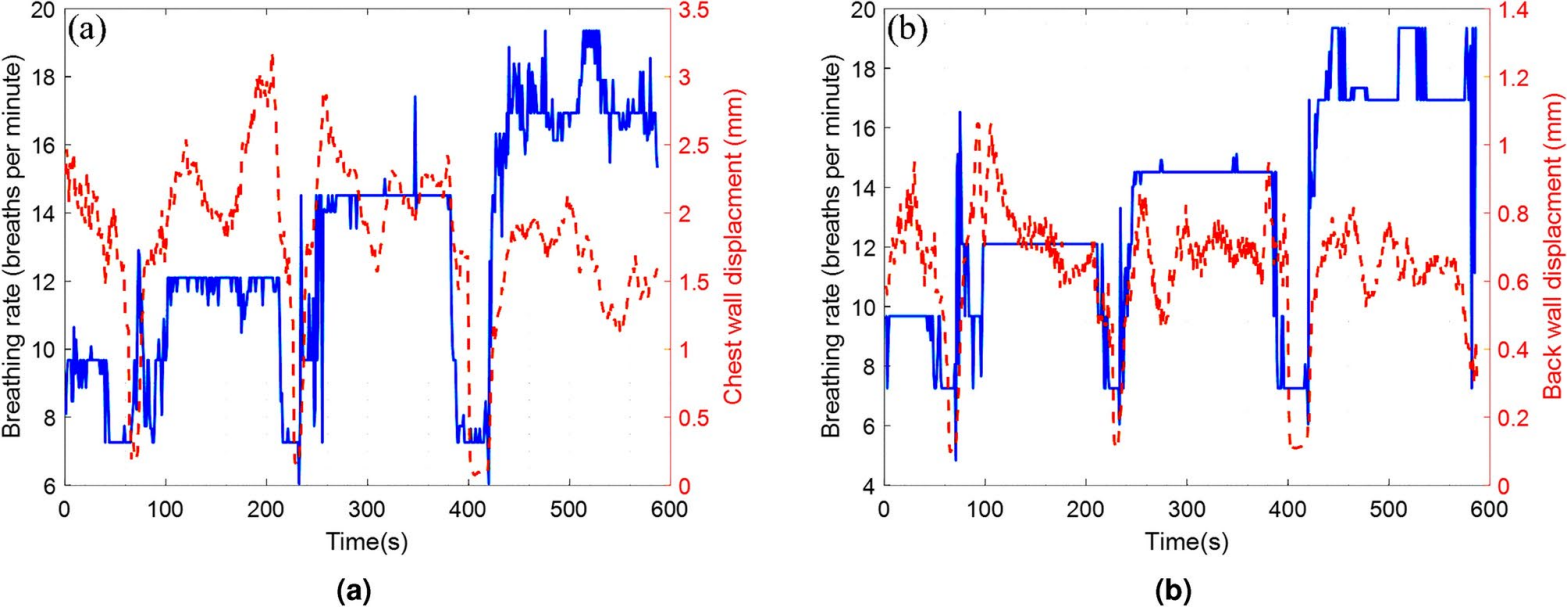
BR estimations were conducted using a dual radar setup at various BRs, including 9, 12, 15, and 17, under conditions where the participant remained without significant body movements: (**a**) desk radar and (**b**) seat radar.

Figure 10a,b represent BR estimation by desk and seat radars, respectively. In this scenario, the participant is asked to follow an online metronome for different BR rates^58^, including 9, 12, 15, and 17, while the participant did not have body movements. Between two different rates, there is a 20 to 30-second breath-hold period in which BR drops during a breath-hold period and displacement is less than 0.15 mm^51^. Table 2 compares the BR estimation errors for both radars. The results indicate that both radars have absolute errors of less than 1.12 breaths per minute while the seat radar is slightly better than the desk radar in various BRs. However, body movements like head and hand movement can affect BR and HR estimations in desk radar more than seat radar. Furthermore, the estimated back wall displacements are less than chest wall displacements. Therefore, they might be affected by phase noise.

**Fig. 11:**
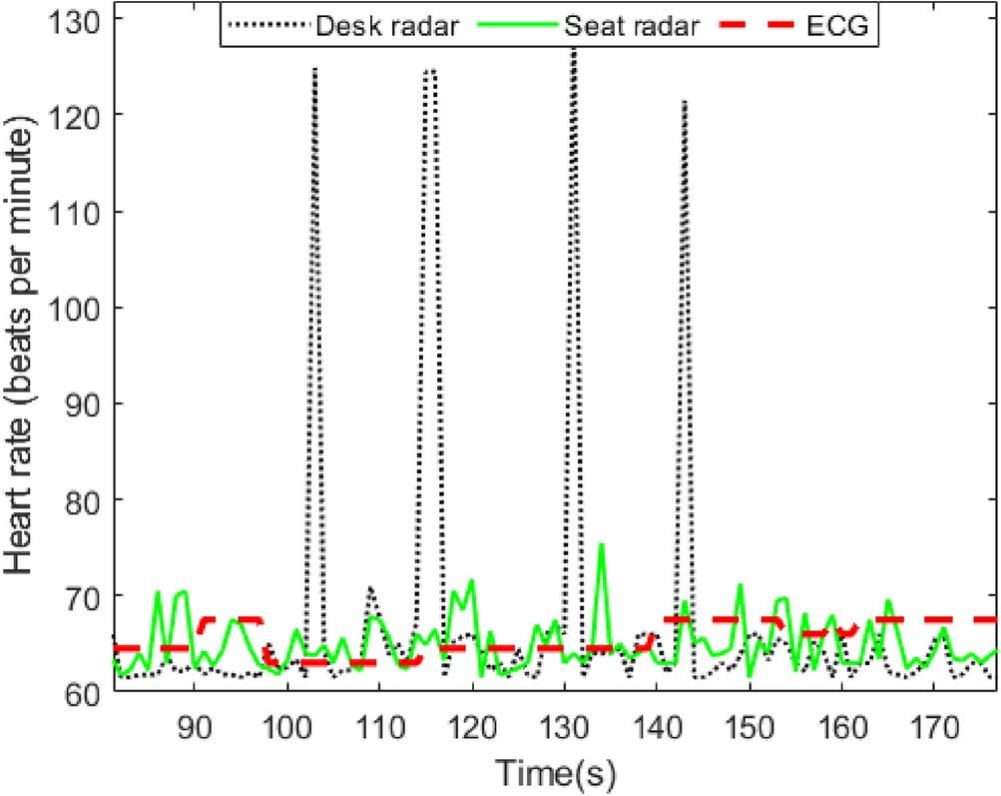
The comparison between the desk and the seat radars estimations for HR estimation based on ECG results.

Figure 11 compares synchronized desk and seat radar results with ECG data for HR estimation. Both radars can track HR when there is no body movement. However, when there are head and hand movements that are quite possible to happen for seated people, the desk radar is affected more than the seat radar. As can be seen in Fig. 11, desk radar estimations for HR spikes incorrectly when there are body movements. However, seat radar is not affected by hand and head movements and can track HR accurately. Root mean squared error(RMSE) for the desk and chair radars are 7.9 beats and 4.1 beats, respectively. However, it cannot compare these two radars in terms of false estimations made by body movements. As a result, using radar behind the seat might reduce body movement effects, in particular head and hand movements.

**Fig. 12:**
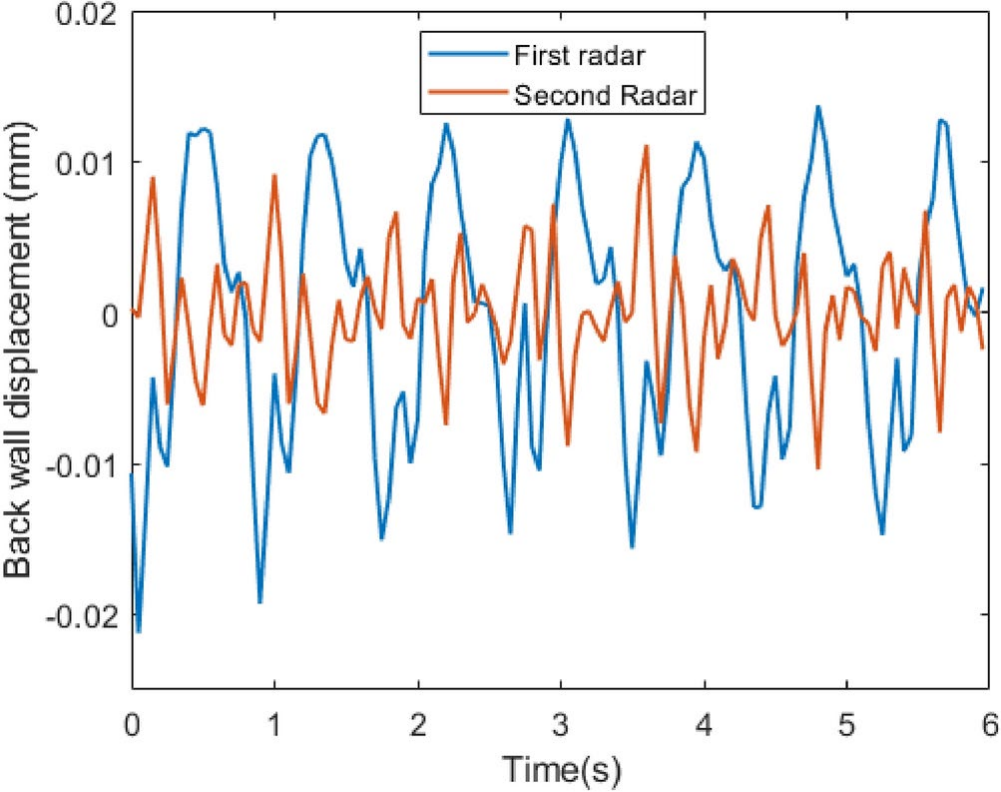
Comparing cardiac waveforms captured by two synchronized radars from different positions, one situated at the heart height and the other behind the participant’s waist.

Figure 12 compares the cardiac waveform resulting from two synchronized radars from behind the seat when there is no interference between them. The first radar is located at the same height as the heart. The second radar is located behind the participant’s waist. The second radar results are almost different from the first radar. Firstly, the valleys in the second radar are not strong enough to be reliably used for HRV estimation and the amplitude of displacement is also less than in the first radar. Secondly, the second radar results are less quasi-periodic. However, the period between the valleys of the first and the second radars remained constant.

**Table 2:** The average absolute errors of BR estimations in different BRs for desk and seat radars.

BR	Desk radar average absolute error (breaths per minute)	Seat radar average absolute error (breaths per minute)
9	1.12	0.81
12	0.35	0.12
15	0.57	0.47
17	0.85	0.65

One of the challenges in using radars is the effect of interference when there are a couple of radars in smart homes. In this paper, the effect of interference is evaluated by a dual radar setup, including the seat radar and the wall radar as can be seen in Fig. 6b. In the first experiment, the wall radar is blocked by a metallic sheet to minimize the effect of this radar on the seat radar while radars are sending signals without a time delay. In the second experiment, a metallic sheet does not block the wall radar and the time delay between radars is also zero. In the final experiment, radars are working with a 5 ms delay while a metallic sheet has not blocked the wall radar. This delay is almost two times the actual timing of a frame in each radar. Therefore, radars send signals separately in the time domain to have less interference effect. The average power in zero doppler in the second experiment is 0.7 dB greater than in the first experiment. However, the power difference between the first and the last experiments is almost zero. As a result, this time delay configuration can solve interference issues in smart home monitoring.

### Validation of the proposed algorithm for health monitoring using FMCW radar

**Fig. 13:**
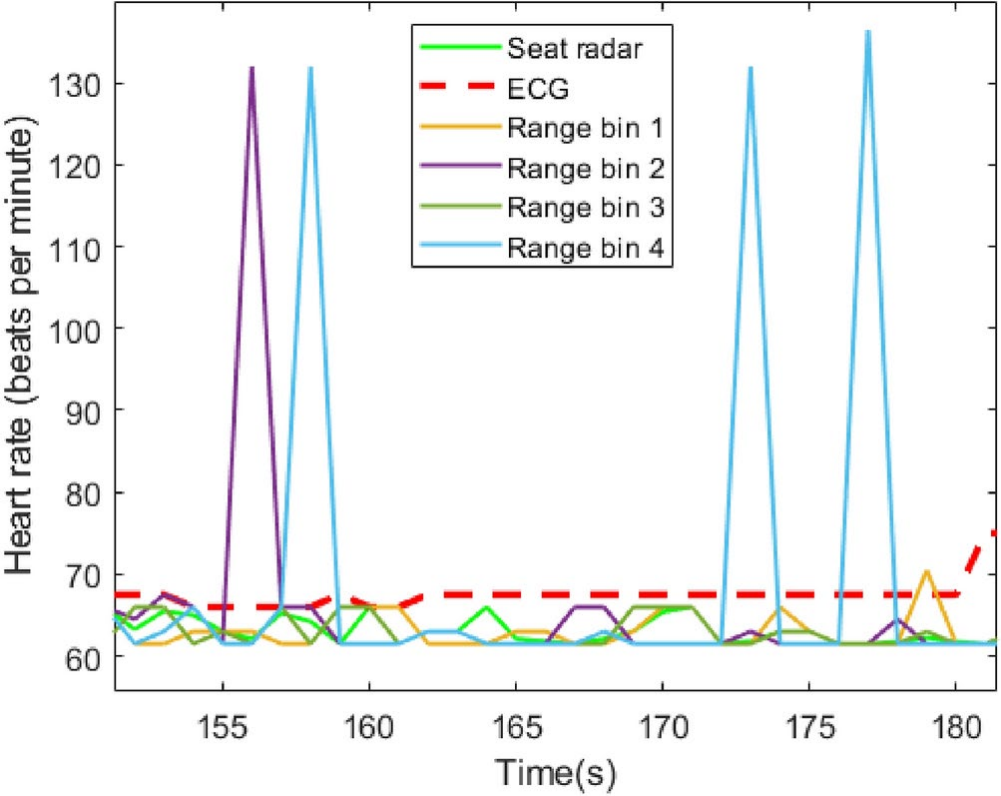
The comparison between the different range bins in the seat radar for HR estimation with ECG.

Figure 13 represents seat radar results in different range bins for HR estimation. The seat radar estimation is based on these 4 range bins. Therefore, false estimations might be removed by using an outlier removal algorithm. However, using a single range bin could be affected by random body movements. Since, the human body captures more than one range bin when the radar is close to the human body, especially behind the seat, using multiple range bins can improve HR and cardiac waveform estimations.

**Fig. 14:**
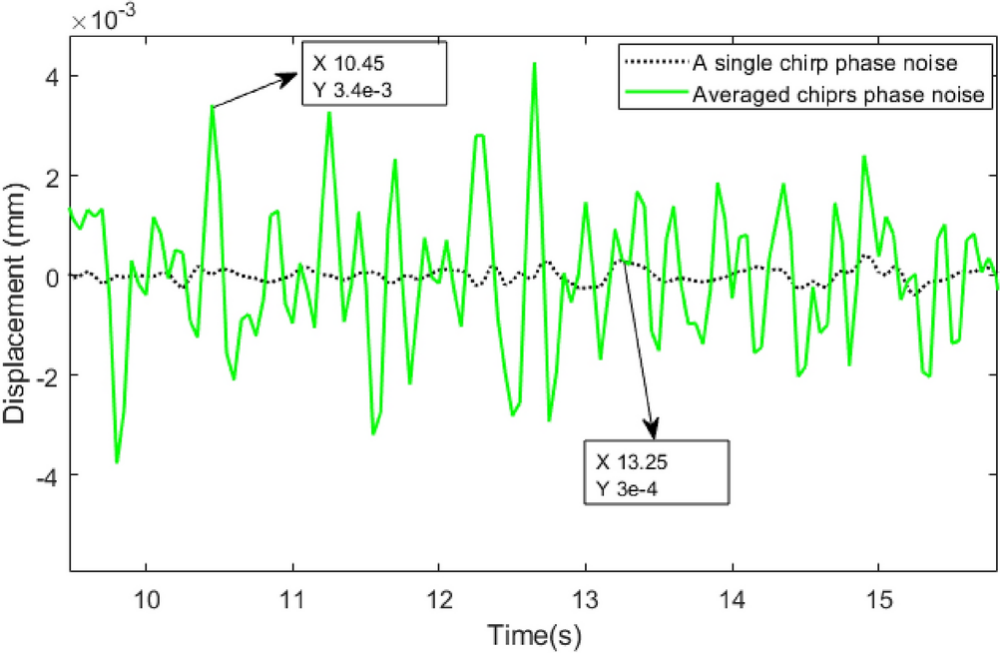
Comparison between phase noise of a single chirp and averaged chirps in a frame with 32 chirps.

Figure 14 compares the phase noise of a single chirp and averaged chirps in a frame. By taking an average of 32 chirps in a frame, the phase noise is suppressed by almost 10 dB. Since breathing displacements are much stronger than phase noise, using a single chirp can estimate displacements^5^. However, the cardiac waveform might be affected by the phase noise of a single chirp. As seen in Fig. 11, heart displacements reach 0.02 mm which could be affected by phase noise. As a result, using an average of multiple chirps in a frame can effectively improve cardiac waveform estimations.

**Fig. 15:**
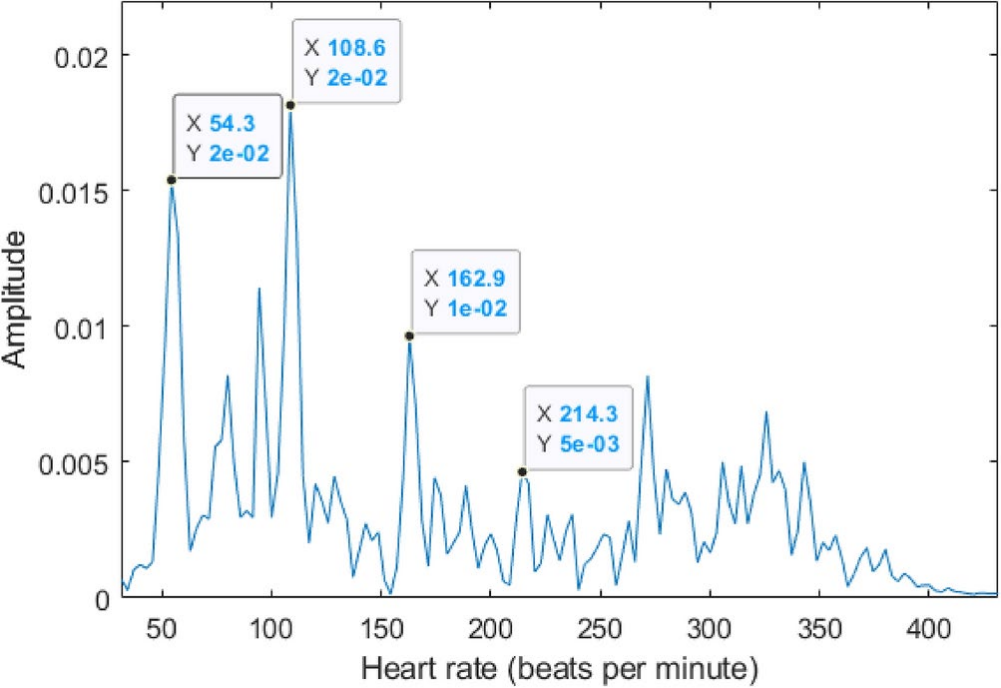
The heart displacements in the frequency domain during the breath-hold while the lower and upper cut-off frequencies of the bandpass filter are 1 Hz and 6 Hz, respectively.

**Fig. 16:**
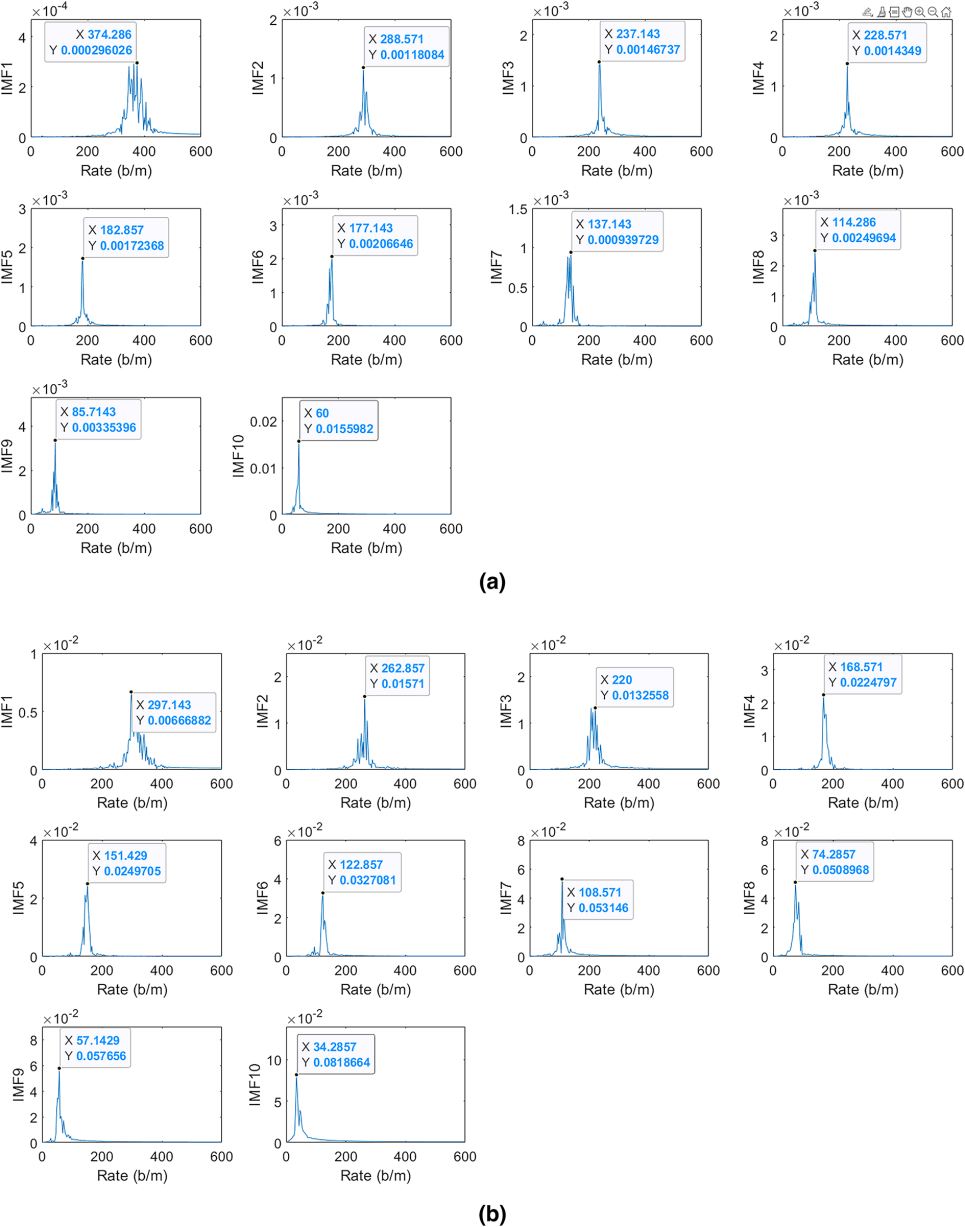
Different IMFs results while the BR was 17 breaths per minute in two distinct breathing patterns: (**a**) shallow breathing and (**b**) deep breathing. b/m stands for beats per minute.

Figure 15 depicts the heart displacement harmonics of a participant during a breath-hold while radar is located behind the seat at almost the same height as the heart. The lower and upper cut-off frequencies of the bandpass filter are 1 Hz and 3 Hz, respectively^59^. The estimated HR based on the radar results is 108 beats per second. However, the actual HR based on the ECG data is 55 beats per minute. The estimated HR by radar is two times of actual value. Therefore, the first peak, which is the actual HR is suppressed by the rising edge of the bandpass filter. As a result, it is necessary to reduce the lower cut-off frequency of the bandpass filter. In this paper, we applied the bandpass filter from 0.8 to 6 Hz.

Figure 16a,b show different IMF results while the participant had shallow and deep breathing, respectively, with a BR of 17 breaths per minute. In shallow breathing, the strongest IMF, which is also the lowest frequency component results from heart displacements. As a result, the HR is 60 beats per minute. In deep breathing, the strongest IMF which is IMF10 results from the second harmonic of breathing vibrations since it is two times of actual BR. IMF 9 is the main harmonic of heart displacements. Therefore, the HR is 57 beats per minute.

**Fig. 17:**
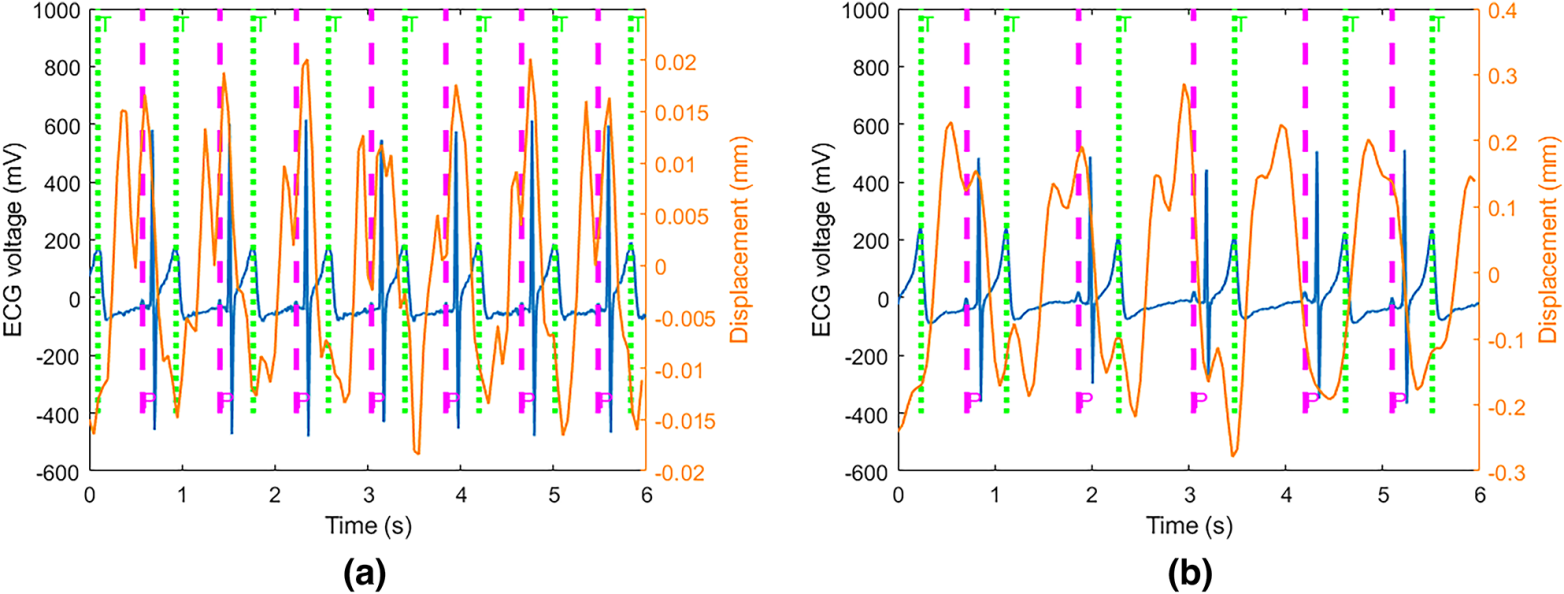
The reconstructed cardiac waveform based on the harmonic analysis compared with ECG data while the BR was 17 breaths per minute in two distinct breathing patterns: (**a**) shallow breathing and (**b**) deep breathing.

**Fig. 18:**
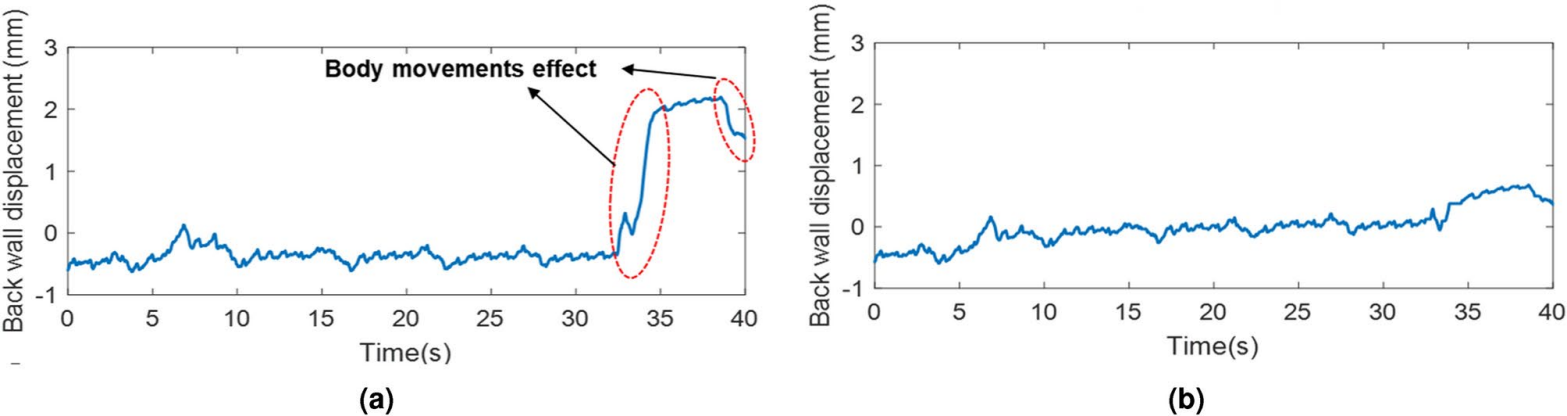
Displacements from behind the seat when radar has the same height as the heart: (**a**) before body movement compensation. (**b**) After body movement compensation.

**Fig. 19:**
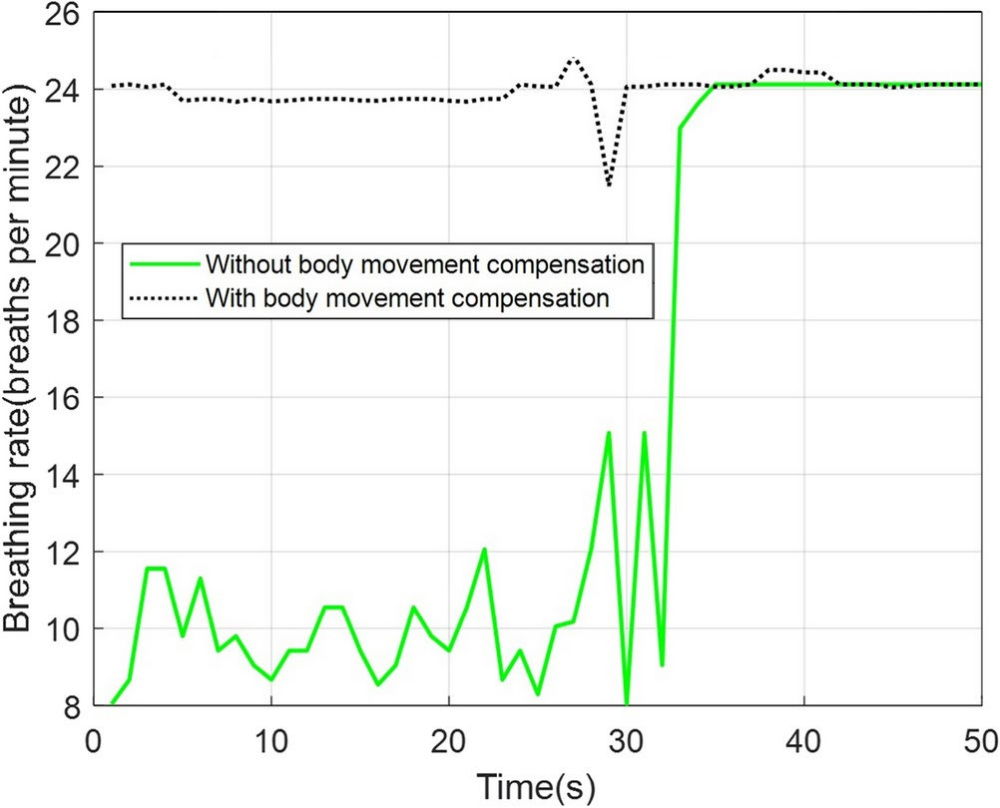
BR estimation when actual BR is 24 from behind the seat before and after body movement compensation.

**Table 3:** The average absolute errors of BR estimations in different BRs during the body movements.

BR	Average-error (breaths per minute)
9	0.9
11	1.1
14	2.9
16	5.7
18	8.1
22	11.5
24	13.2

**Fig. 20:**
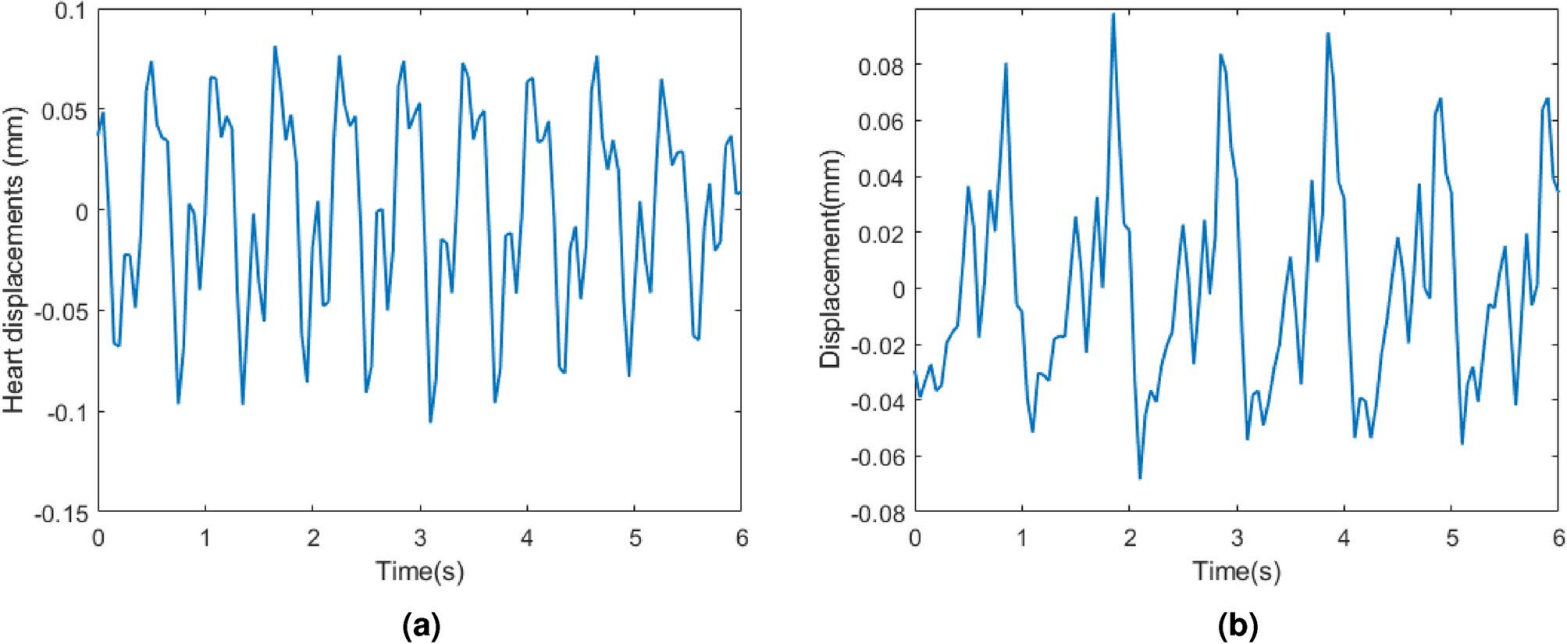
The cardiac waveform of two older adult subjects with two different heart conditions: (**a**) Tachycardia condition and (**b**) prolonged QTc.

However, the cardiac waveform can be reconstructed by using multiple harmonics. In shallow breathing, the second harmonic of heart displacement is 120 beats per minute. Since this rate is not among the detected IMFs, it might be distorted by other harmonics. The third and fourth harmonics’ rates are 180 and 240 respectively. IMF 5 and IMF 3 correspond for the third and the fourth harmonics rate respectively. The reconstructed cardiac waveform based on this harmonic analysis is shown in Fig. 17a. The valleys can be aligned with T-waves. However, the waveform does not have two sequential peaks all the time similar to the breath-hold period. In deep breathing, the second harmonic of heart displacements interfered with other harmonics and cannot be recovered. However, the third harmonic of heart displacements is IMF4. The reconstructed cardiac waveform based on these harmonics is shown in Fig. 17b. The reconstructed cardiac waveform has two peaks. However, when valleys align with T-waves, P-waves do not align with the second peak. Therefore, during deep breathing, valleys correspond to T-waves and can be utilized for HRV estimation, like shallow breathing.

Figure 18a,b compare back wall displacements measured by radar from behind the seat before and after body movement compensation. As can be seen, body movements cause significant spikes in a short period. These spikes should be mitigated before cardiac waveform reconstruction. Otherwise, due to the strong amplitude, they corrupt the breathing band and heart band. The proposed approach for body movement detection can effectively detect all body movements. Figure 19 depicts the body movement compensation effect on BR estimation when actual BR is 24. As can be seen, due to the body movements, the estimated BR drops to almost 10 breaths per minute. Table 3 compares the absolute estimation error of BR for different BRs during the body movements. As can be seen, when BR is high, the average absolute error of BR increases significantly.

### Cardiac waveform verification in subjects with heart conditions

Figure 20a depicts the cardiac waveform of an older adult subject with tachycardia condition, resulting in an elevated HR even during periods of rest. As illustrated, the cardiac waveform displays two peaks followed by a single valley in each cycle, with a resting HR of 101 beats per minute. Healthy subjects exhibit a similar cardiac waveform, with a normal HR during rest.

In addition, the first peak is stronger than the second peak which is similar to healthy subjects’ cardiac waveform after physical exercise. Figure 20b illustrates the cardiac waveform of an older adult subject with a prolonged QTc. The depicted waveform deviates from the typical pattern observed in healthy subjects, where two peaks are followed by a valley. In contrast, the subject with a prolonged QTc displays three peaks followed by a valley. This divergence from the norm suggests a potential heart condition. Therefore, variations in the derived cardiac waveform can serve as an indicator of underlying cardiac issues in comparison to the waveform observed in healthy individuals. It is notable that all four subjects with a prolonged QTc have normal HR and HRV in our experiments.

**Fig. 21:**
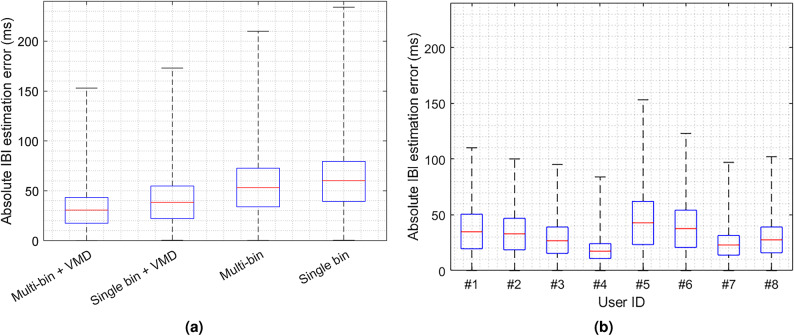
The error in absolute IBI estimation: (**a**) the impact of different signal processing approaches and (**b**) the impact of user diversity.

**Table 4:** The average absolute errors of HR estimations in different participants by the proposed method.

User ID	Relative error (%)
1	5.2
2	5.1
3	4.6
4	2.9
5	5.9
6	5.4
7	4.6
8	4.7

**Table 5:** HRV estimation errors in terms of RMSSD, SDRR, and pNN50 for 8 subjects.

Metrics	User ID							
	1	2	3	4	5	6	7	8
RMSSD (ms)	5.9	6.4	3.4	8.3	7.3	5.6	4.5	7.3
SDRR (ms)	11.2	5.8	3.8	7.6	6.5	7.2	5.3	6.5
pNN50 (%)	6.1	11.1	4.6	6.5	4.5	4.2	6.1	7.3

**Table 6:** The comparison between this study and the recent published papers based on four factors, including body movement compensation, breathing harmonic cancellation, sensitivity to range bin selection, and achieved results.

References	Body movement compensation	Breathing harmonic cancellation	Sensitivity to range bin selection	HRV metrics	HRV metrics error	HR error (%)
^15^	No	No	Yes	NA	NA	NA
^34^	No	No	No	RMSSD	NA	2.42
^38^	No	No	Yes	SDNN, RMSSD, LF, and HF	NA	2.07
^39^	No	No	Yes	NA	NA	1.3
^40^	No	No	Yes	NA	NA	NA
^43^	No	No	Yes	LF and HF	NA	NA
^44^	Yes	Yes	Yes	Mean IBI, RMSSD, SDRR, pNN50	3.9 ms, 6.4 ms, 6.4 ms, 2.6%	NA
^37^	Yes	Yes	Yes	SDNN, RMSSD	NA	NA
^41^	Yes	Yes	Yes	SDNN	6 ms	3
This study	Yes	Yes	No	RMSSD, SDRR, pNN50	4.5 ms, 6.1 ms, 6.7 ms, 6.3%	4.8

## Discussion

Figure 21a compares the impact of different signal processing methods on the absolute IBI estimation error. The combination of the multi-bin approach with VMD demonstrates a median error reduction of approximately 20 ms compared to the multi-bin approach without VMD. Notably, the maximum error also exhibits significant improvement, with a reduction of 57 ms. Additionally, the utilization of multiple bins also directly influences both the median and maximum IBI estimation errors. When VMD is also applied, there is an enhancement of approximately 8.2 ms in the median error and 20 ms in the maximum error. Furthermore, the study’s outcomes show a median IBI estimation error of 30 ms. These findings underscore the effectiveness of the proposed method in this paper.

To evaluate the proposed method’s robustness across diverse users, Fig. 21b depicts a comprehensive illustration of the distribution of absolute errors in IBI estimation for all eight participants. The findings reveal that our proposed method yields varying IBI estimation errors among different users from 17.5 to 42.8 ms, with an overall 95% confidence interval of [3.0075 ms, 65.2946 ms]. These variations can be attributed to a range of factors, including differences in individual physiological traits, such as body mass and variations in cardiac strength. Importantly, it is evident that the 75th percentile error for all participants consistently remains below 62 ms. In addition, Table 4 also compares the absolute estimation error of HR for all participants. The results indicate that the absolute relative error for HR estimation reached 4.8%. To assess the accuracy of HRV estimation, Table 5 presents the estimated HRV metrics, including RMSSD, SDRR, and pNN50 for 8 participants. The results indicate that the proposed method achieves an average error of 6.1 ms for RMSSD, 6.7 ms for SDRR, and 6.3% for pNN50. These observations underscore the method’s resilience and effectiveness when applied to a diverse group of subjects.

Table 6 compares this study with recent publications across four criteria: body movement compensation, breathing harmonic cancellation, range bin sensitivity, and resultant outcomes. Emphasizing the necessity of addressing body movements and breathing harmonics in signal processing for realistic applications, the study highlights their significant impact on estimated vital signs. Additionally, the stability of radar integrated into furniture provides resilience against human manipulation, suggesting a reduced necessity for specific range bin selection in signal processing for the radar positioned behind the seat. The study’s outcomes show reliable results based on HRV metrics and HR errors.

## Conclusions

In this investigation into cardiac health monitoring within smart furniture using a mm-Wave radar, our study reveals distinctive cardiac waveform patterns for healthy individuals, even during breathing, when the radar is strategically positioned behind the seat. This specific pattern includes two peaks and a valley in each cycle offering reliable HRV estimation, particularly with the valleys. Additionally, the cardiac waveform extracted from individuals with prolonged QTc is compared to that of healthy individuals. The comparison reveals distinguishable differences, indicating that those with this condition exhibit a different cardiac waveform compared to their healthy counterparts. Consequently, this distinctive cardiac waveform serves as a potential indicator for the detection of underlying heart issues. As a result, this system provides a low-power, cost-effective, and non-contact solution for monitoring heart issues in elderly individuals who may forget to wear sensors, all while ensuring privacy. The proposed signal processing chain enables accurate IBI and HR estimation, with a median absolute error of approximately 30 ms for IBI and an average relative error of 4.8% for HR estimation, with an overall 95% confidence interval of [3.0075 ms, 65.2946 ms]. Furthermore, the study addresses seated individuals’ body movements using a time-domain approach, effectively detecting and mitigating these movements.

## Data Availability

The datasets used and/or analyzed during the current study are available upon request. For more information, please contact George Shaker at gshaker@uwaterloo.ca.
